# Anatase Forming Treatment without Surface Morphological Alteration of Dental Implant

**DOI:** 10.3390/ma13225280

**Published:** 2020-11-22

**Authors:** Saturnino Marco Lupi, Benedetta Albini, Arianna Rodriguez y Baena, Giulia Lanfrè, Pietro Galinetto

**Affiliations:** 1 Department of Clinical Surgical, Pediatric and Diagnostic Sciences, University of Pavia, 27100 Pavia, Italy; giulia.lanfre01@universitadipavia.it; 2 Department of Physics, University of Pavia, Via Bassi 6, 27100 Pavia, Italy; benedetta.albini01@ateneopv.it (B.A.); pietro.galinetto@unipv.it (P.G.); 3 Department of Dentistry, IRCCS San Raffaele Hospital, Dental School, Vita Salute University, 20132 Milan, Italy; arianna_rodriguez@hotmail.it

**Keywords:** anatase, titania, dental implant, Raman spectroscopy

## Abstract

The osseointegration of titanium implants is allowed by the TiO_2_ layer that covers the implants. Titania can exist in amorphous form or in three different crystalline conformations: anatase, rutile and brookite. Few studies have characterized TiO_2_ covering the surface of dental implants from the crystalline point of view. The aim of the present study was to characterize the evolution of the TiO_2_ layer following different surface treatments from a crystallographic point of view. Commercially pure titanium and Ti-6Al-4V implants subjected to different surface treatments were analyzed by Raman spectroscopy to evaluate the crystalline conformation of titania. The surface treatments evaluated were: machining, sandblasting, sandblasting and etching and sandblasting, etching and anodization. The anodizing treatment evaluated in this study allowed to obtain anatase on commercially pure titanium implants without altering the morphological characteristics of the surface.

## 1. Introduction

Ever since it was proposed by Brånemark in the 1960s [1], titanium has been considered the best material for the production of endosseous anchorages and dental implants, although it is not the only one [2,3,4]. Titanium dental implants have demonstrated high survival and success rates in both the short and long term [5,6]. The biocompatibility, passivation, and chemical resistance characteristics of the rapidly forming titania (TiO_2_) layer on the metallic bulk are the basis of the clinical success of the titanium implants [3,7,8,9].

The TiO_2_ layer has a thickness of between 3 and 40 nm on dental implants [10].

The native TiO_2_ formed during normal ambient conditions on Ti is amorphous if there is no additional treatment. In addition to the amorphous phase, three different crystalline polymorphs of titania exist naturally, namely anatase, rutile, and brookite, with the last being rarely reported [11,12,13].

Among the three conformations, rutile is thermodynamically the more stable phase at ambient temperature and pressure [14], but anatase is kinetically more stable and consequently is the more common phase for the formation of nanocrystalline titania at relatively lower temperature [13].

Soft chemistry method, hydrothermal process, and the sol–gel route allow to obtain titania [15]. Since there is no equilibrium between the polymorphs of titania, there is no specific temperature for the phase changes to occur [13].

Usually, the transformation from the amorphous phase to anatase occurs between 400 and 550 °C, from anatase to rutile between 600 and 1100 °C [13,16,17]. Nevertheless, the three possible crystalline forms could be subjected to phase transformation to one another when processed to particular physicochemical conditions and depending also on the nanometric morphologies of the samples [18,19].

Titania is well known for catalytic, photocatalytic and nonlinear optics properties [17,20,21]. While the effects of the morphological and chemical characteristics of TiO_2_ have been extensively studied [22,23], few studies evaluated the biological effects of the crystalline conformation of titania [12]. Apatite deposition is enhanced by anatase [11,24,25,26,27,28]. Anatase enhance osteoblast activity in vitro [29]. Anatase and rutile coated implants showed an increased osseointegration in vivo [29,30]. Moreover, anatase coating can inhibit bacterial adhesion on titanium surfaces [31,32,33,34,35]. Since pure titanium does not have adequate mechanical characteristics, two titanium-based materials are mainly used for the production of dental implants: grade 4, also called commercially pure titanium (CP), and grade 5 (TA), which consists of an alloy of titanium, aluminum and vanadium (Ti-6Al-4V). In their composition both CP and TA have traces of N, C, H, Fe, and O (see Table 1 in the experimental section). The composition of TA consists of about 10% by weight of Al and V (Table 1) [36].⁠ While there are many studies evaluating the effects of surface treatments on the morphological and chemical characteristics of titanium implants [23,37], there are few studies in the literature evaluating the presence of crystalline titania on the surface of dental implants [12,38].

Thermal treatment and anodization (anodic oxidation) are recognized as capable of producing a layer of crystalline titania on the surface of CP and TA [25,30,39,40]. Some recent studies have confirmed the presence of anatase on commercially available implants with anodized surface [41,42].

Atmospheric pressure plasmas have demonstrated to effectively reduce carbon contamination and enhance free energy of implant surfaces [43,44,45].

Recently, our group analyzed the effect of different surface treatments on the crystallinity of the TiO_2_ layer of TA (Ti-6Al-4V) implants [46]. The study showed that the increase in the complexity of the surface treatment corresponded to an increase in the thickness of the amorphous oxide layer without obtaining crystalline TiO_2_. Furthermore, the sandblasting treatment with corundum caused a contamination of the final surface with traces of Al_2_O_3_.

Raman spectroscopy (RS) is a well-established method to monitor titania formation and its crystal habit due to the great sensitivity to structural changes and due to the higher Raman efficiency of Ti-based oxides. In addition, RS is non-invasive and non-destructive, and thus it is widely used in different basic and applied research fields involving chemically and mechanically processed functional oxides [47,48,49,50].

The present study, therefore, was aimed to monitor with micro-Raman spectroscopy the evolution of the TiO_2_ layer from the crystallographic point of view of implants obtained starting from different materials, i.e., CP titanium (grade IV) and TA (Ti-6Al-4V) and subjected to different surface treatments. The structural analyses by Raman spectroscopy has been corroborated by complementary analyses using UV and SEM microscopies. SEM-EDX measurements have been also performed allowing an elemental quantification as a confirmation of the structural information derived by Raman inspection. Thus, with this approach, we obtain an estimation of the oxide layer and of Al_2_O_3_ contamination.

## 2. Materials and Methods

To characterize the crystallographic transformation of TiO_2_ induced by manufacturing phases, experimental implants made of CP and TA were specifically produced by FMD Medical Devices (Rome, Italy) for the present study.

The compositions of the pristine materials according to factory datasheets [36] and to the international standards are reported in Table 1.

For the observations, screw-shaped implants with threads 5.5 mm wide and 14 mm long were produced. All the implants were obtained with manufacturing procedures commonly used in the production of commercial implants and were produced starting from the same batch of TA and CP.

Each implant characterized by a single surface was produced in duplicate. Each implant was obtained through a procedure, consisting of: (i) machining (milling) of the raw material in the presence of a lubricant, (ii) ultrasonic cleaning with solvents, that was the same for all and then subjected to the final surface treatment. After the surface treatment, all implants were subjected to packaging in glass jars under laminar flow hood and dry heat sterilization for 3 h at 170 °C.

The samples differed only in the final surface treatment. The surface treatments evaluated were: machining, sandblasting, sandblasting and etching and sandblasting, etching and anodization.

The sandblasting was carried out with Al2O3 particles with an average size of 180–200 μm. The etching was carried out for 15 s with an HF (0.5%) and HNO3 (64.5%) solution. The anodization was carried out with direct current anodization for 15 s at 60 V. Each surface treatment was conducted on CP and TA implants. The surface treatments evaluated in this study were chosen as they are commonly used for the production of commercially available implants.

### 2.1. Raman Spectroscopy

An Olympus HS BX40 (Shinjuku Monolith, Tokyo, Japan) microscope equipped with a LabRAM Dilor (HORIBA, Kyoto, Japan) spectrometer were used to conduct micro-Raman measurements at room temperature. The working parameters for Raman spectrometry as well the experimental procedures and data best-fitting procedures were the same used in our previous study [46]. Here, we recall the main experimental elements: (i) the sampled area during the measurements was around 4 µm^2^ and the power density on the samples of the 632.8 nm laser spot was about 5 × 105 W/cm^2^, (ii) particular care was paid to the measurement of background signals for reliable subtractions for each sample; (iii) the best fitting procedures were performed by using Lorentzian curve as fitting function, and (iv) typical integration times were about 2–3 min and the mapping measurements required approximately 2 h of counting.

All the implants were analyzed in two different regions, i.e., on the top of the screw and on the middle of the screw. These two regions were chosen to evaluate whether the surface treatments (especially sandblasting) were in any way influenced by the presence of the threads of the screw.

### 2.2. UV Fluorescence Microscopy and SEM/EDS

All the samples were observed under the optical microscope (Olympus SZ61, Olympus Corp., Tokyo, Japan) on the top of the screw with three different magnifications, i.e., 100×, 200× and 500× equipped with digital camera Infinity1 (Olympus Corp., Tokyo, Japan); then were observed under UV fluorescence microscope (Olympus BX51, Olympus Corp., Tokyo, Japan) with 500× magnification, with an Olympus UC30 (Olympus Corp., Tokyo, Japan) digital camera.

In a second measuring session, all the listed samples were analyzed by a Scanning Electron Microscope (MIRA3, TESCAN ORSAY HOLDING, a.s., Kohoutovice, Czech Republic) providing also EDS elemental analyses. For A1 and T1 samples only a micrograph at 500x magnification was collected, as they are the starting samples not yet processed with a surface treatment. For all the other samples a first image at 160× magnification was collected, then a second one at 500× magnification and after that micrographs were recorded in the same area at increasing magnifications (1 k×, 2 k× 1 k×, 2 k×, 5 k×, 10 k×, and 25 k×).

The SEM images were collected both using secondary electrons (SE) and the back scattered electrons (BSE) one. The first provides for morphology of the sample surface with an image contrast depending on the roughness and orientation of the surface, while the second allows to distinguish between areas with different chemical composition, due to the sensitivity to the atomic weight of the specimen elements [51,52].

An estimation of the thicknesses of the TiO_2_ layers was derived by calculating the ratio between the percentage of surface O and Al obtained through EDS analysis.

The whole set of investigated samples, with proper codes, as well as indications about pristine materials and treatments is reported in Table 2.

## 3. Results

### 3.1. Optical and UV Microscopy

**Figure 1:**
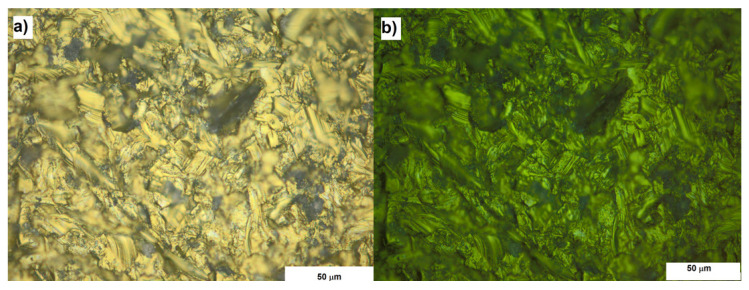
Microphotography of sample CP4 representative of all surfaces subjected to sandblasting. (**a**) Optical (**b**) UV fluorescence. Magnification 500×.

The optical and UV microscopy allowed to analyze the homogeneity of the surfaces. From the morphological point of view, at this spatial resolution degree, no any particular differences have been observed. The UV microscopy revealed the presence of photoluminescent signal, associated to the corundum contamination, from all the sandblasted samples. Anyway, all the surfaces subjected to sandblasting had the same appearance both under UV light and optical microscopy (Figure 1).

### 3.2. Raman Spectroscopy

**Figure 2:**
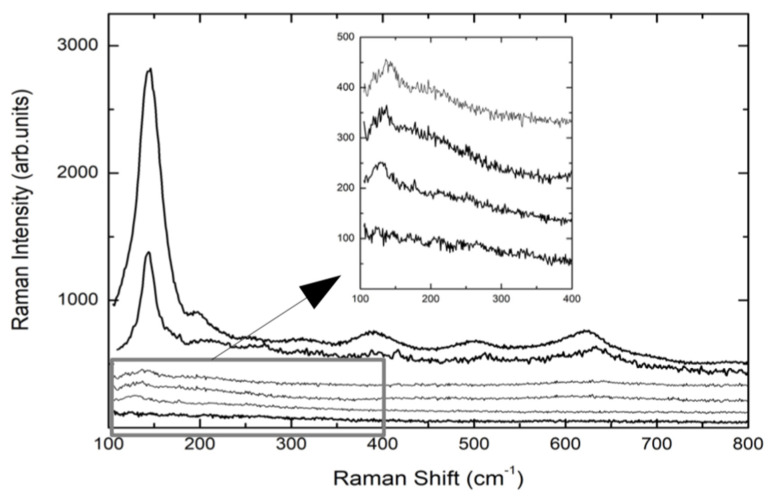
Raman spectra obtained from TA and CP implants. From the bottom: CP1/TA1, TA3, TA4, TA4p, CP3, CP4. In the inset the low energy region for the TA samples is reported in order to give evidence to the Raman signal at around 145 cm^−1^.

Figure 2 reports the Raman spectra of the implants reported in Table 2, with the exception of TA1 and TA2, whose Raman spectra are completely equal to that of CP1 sample. 

**Figure 3:**
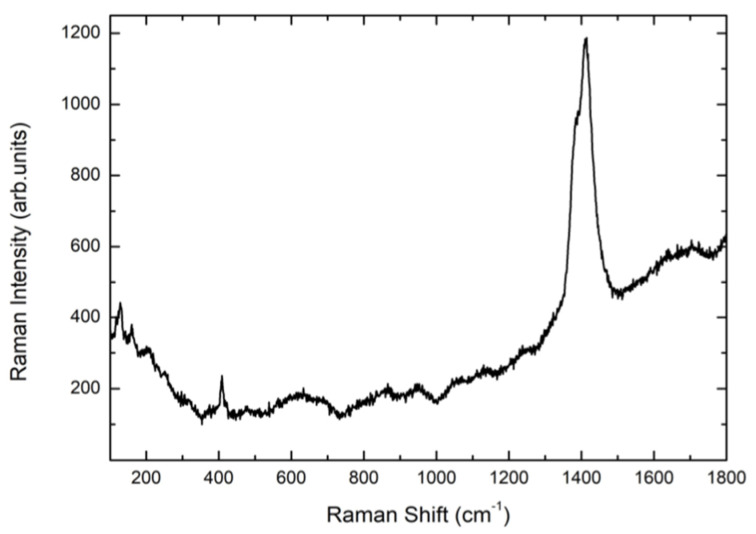
The Raman spectrum of CP3 sample in the region between 100–1800 cm^−1^ with the strong PL signal associated to Cr^3+^ impurities (see text).

The spectra are the result of different experimental runs in different regions of the top of the screws and from the middle. The Raman signals from TA screws are characterized by a very low Raman yield with a weak peak detectable at around 143 cm^−1^, indicating the formation of a very disordered TiO_2_ layer. Even the anodization treatment (TA4) provokes a very small increase of the Raman signal of the A1g mode of Anatase. The same happens when the TA-based screw is further processed by plasma treatment (TA4p). These results from TA implants are the confirmation of what we found in previous observations [46]. Different behavior was observed for CP-based screws. Indeed, the Raman spectrum of CP3 sample, i.e., CP-based screw treated by Al_2_O_3_-blasting and HNO_3_/HF etching, presents a well-defined and intense mode at 145 cm^−1^ and weaker but measurable additional Raman modes at around 400, 510, and 635 cm^−1^. This set of Raman signals represents the Raman fingerprint of anatase TiO_2_. The subsequent anodization treatment, CP4 sample, provokes an increase in the whole Raman yield. The previously reported signal is higher and very well defined indicating a net increase of the crystalline order, which in any case is clearly already present in CP3 sample, i.e., the sample not yet anodized. The crystalline order in processed CP-based screws is missing in processed TA-based implants. For all the samples, the energy region till 1800 cm^−1^ was monitored. Figure 3 shows the Raman signal from CP3 sample acquired in the region 100–1800 cm^−1^.

A strong signal is peaked at 1390 cm^−1^ with a doublet structure. This is the unmistakable fluorescence signal of the Cr^3+^ ion excited by the red light of HeNe laser. As well-known Chromium impurities are usually present in the corundum habit. On the other hand, the fact that Cr^3+^ luminescence is correlated with the presence of corundum is confirmed looking at the lower energy region where the Raman signal from the corundum system Al_2_O_3_ is observed at around 416 cm^−1^. 

One has to observe that the Cr^3+^ luminescence signal has a very high cross-section when enter as substituent of Al ions in the octahedral coordination sites of the crystal lattice of corundum. Indeed, it is well known that even Cr impurity amount of the order of few ppm can give photoluminescence signals quite stronger than those observed by us [53].

On the bases of single point measurements and looking at sample regions where no photoluminescence was present, we have performed different Raman mapping tests in order to monitor the homogeneity of anatase TiO_2_ layers in different screws.

**Figure 4:**
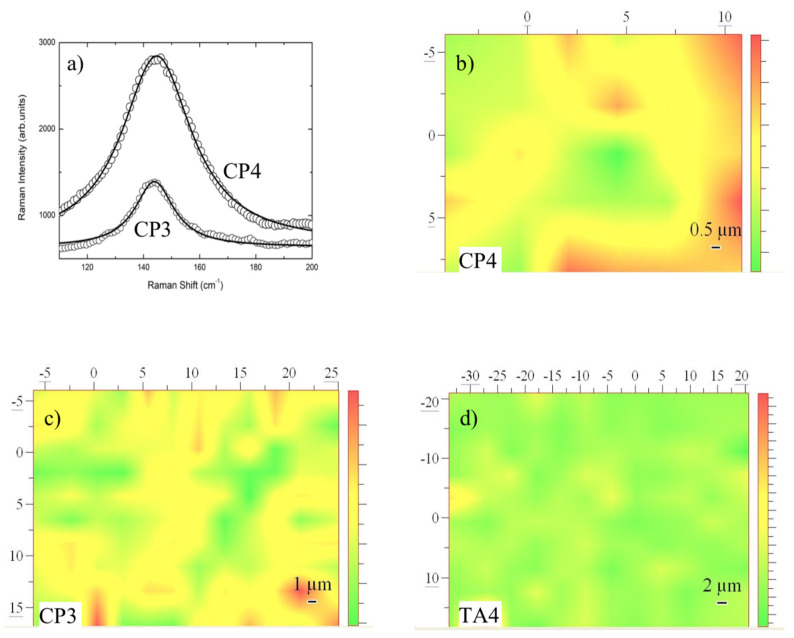
(**a**) Raman spectra in the region 100–200 cm^−1^ for CP3 and CP4 samples (open circle) and the best fitting results (solid lines). The integrated intensities from best fitting procedures have been used to obtain: (**b**) Raman mapping of a region of 15 × 15 μm^2^ of CP4; (**c**) Raman mapping of a region of 32 × 22 μm^2^ of CP3; (**d**) Raman mapping of a region of 55 × 40 μm^2^ of TA4.

The mapping has been obtained using as the intensity marker the integrated intensity of the anatase mode at 145 cm^−1^. Two representative cases are reported in Figure 4a for CP3 and CP4 samples. The Raman mapping allowed to give a pictorial representation of the occurrence and homogeneity of anatase TiO_2_ phase and in turn represents an indication of the thicknesses of anatase titania formed on the screws. In Figure 4b–d the resulting Raman mapping for CP3, CP4, and TA4 are reported.

### 3.3. SEM Images

**Figure 5:**
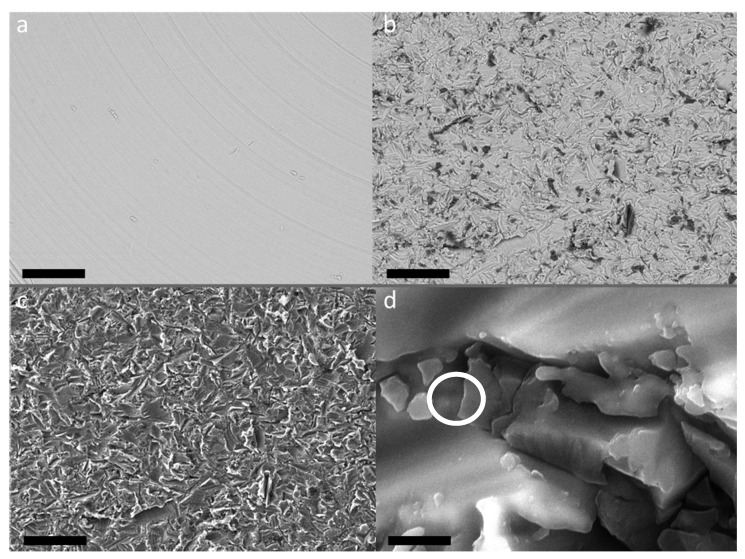
SEM images. (**a**) CP1, BSE detector, 500×, bar scale 100 μm; (**b**) CP4, BSE detector, 500×, bar scale 100 μm; (**c**) same area of b taken with SE detector, 500×, bar scale 100 μm; (**d**) CP4, SE detector, 25,000×, bar scale 2 μm. The grain evidenced by the white circle has been used to obtain EDS spectrum reported in Figure 6.

Important complementary information has been obtained by SEM measurements. In Figure 5a, the BSE micrograph of TA1 sample, i.e., the machined surface of TA implant, is shown.

It is possible to appreciate a smooth and bright surface with the presence of grooves due to the turning and smoothing process of the surface.

The surfaces subjected to sandblasting with Al_2_O_3_ showed a similar appearance regardless of further treatments (Figure 5b–d). This treatment gives rise to a strongly irregular surface, with indented and sharp grooves and ridges.

**Figure 6:**
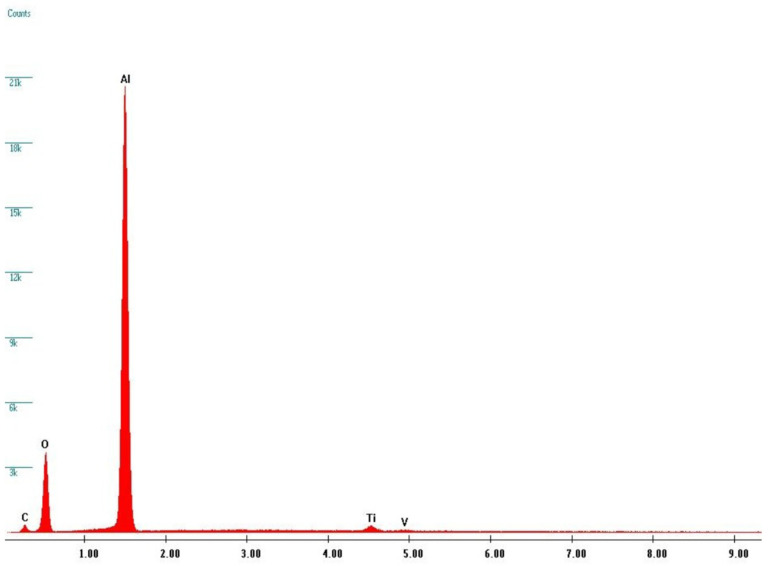
EDS spectrum from SEM analyses on CP4 sample from the grain evidenced in Figure 5d by a white circle. The x-axis unit is Energy (keV).

By comparing the BSE images of the CP1 (Figure 5a) and CP4 (Figure 5b) samples, it is possible to notice, in the last sample, the presence of dark areas within the bright matrix which are not observable in the first sample. Furthermore, at high magnification it is possible to appreciate the presence of small particles embedded in the irregular texture of the titanium and/or titania surface (Figure 6d). Thanks to EDS analyses (see Figure 6), it was possible to verify that these dark areas can be ascribed to corundum micro-grains, present in the screw surface as residual products of the sandblasting process, while the bright matrix is manly composed of Titanium. Indeed, X-ray fluorescence peaks from Al and O are largely prevalent.

The EDS data allow to obtain complementary but quantitative information about elemental distribution among different samples. A detailed analysis has been made moving from the starting compositions of pristine materials and thus using the EDS data from TA1 and CP1 samples as our standards for sake of comparison. We considered for all the samples the ratio between oxygen and aluminum. Indeed, this ratio reflects the amount of oxide formation not correlated to corundum residuals thus depending just from titania formation.

**Figure 7:**
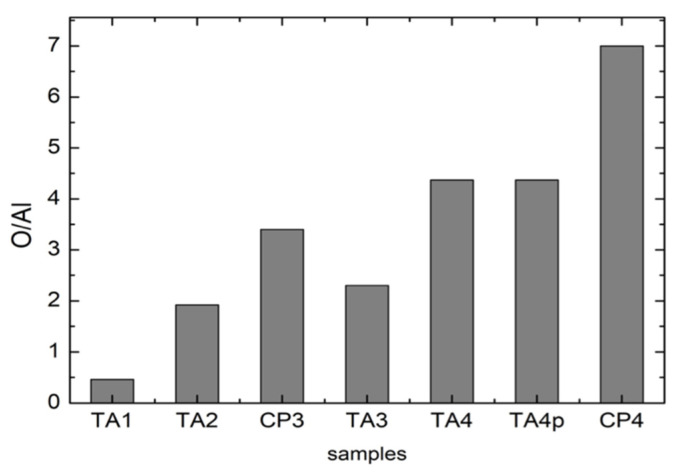
O/Al ratio in all the investigated samples. This ratio has been derived by analyzing EDS data.

The results of these analyses are reported in Figure 7: it is possible to appreciate an increase in the Oxygen amount from TA1 to CP4. This could be associated to an increase in the thickness of the superficial TiO_2_ layer as a result of the manufacturing processes. It can be ruled out that such an increase is due to the residual corundum phase because it has been verified that the oxygen percentage in a single corundum grain is almost the same for all the samples.

One has to notice that the parameter for the untreated CP1 sample has not been derived due to the absence of Al. In addition, the values obtained for TA samples have been derived taking into account the starting amount of Al in pristine TA materials. As can be observed for samples processed in the same manner the values are higher for CP samples. For CP4 sample, we obtained the highest amount of oxygen. This fact seems to strictly correlate with the results from Raman measurements where CP4 exhibits a very well structured anatase Raman spectrum.

It is possible to appreciate an increase in the Oxygen amount from TA1 to CP4; this could be associated to an increase in the thickness of the superficial TiO_2_ layer as a result of the manufacturing processes. It can be ruled out that such an increase is due to the residual corundum phase because it has been verified that the oxygen percentage in a single corundum grain is almost the same for all the samples.

One has to notice that the parameter for the untreated CP1 sample has not been derived due to the absence of Al. In addition, the values obtained for TA samples have been derived taking into account the starting amount of Al in pristine TA materials. As can be observed for samples processed in the same manner the values are higher for CP samples. For CP4 sample, we obtained the highest amount of oxygen. This fact seems to strictly correlate with the results from Raman measurements where CP4 exhibits a very well structured anatase Raman spectrum.

## 4. Discussion

The results of this study confirm the absence of crystalline TiO_2_ on TA implants subjected to sandblasting and double acidification and/or anodization, as found in the evaluations previously reported [46]. The data of the literature are conflicting, since in some cases the presence of anatase or crystalline TiO_2_ has been observed, while in others it has been excluded. Regarding this point, it is important to stress the concept that although in many studies the implants have undergone treatments that can generally be defined as sandblasting, acidification (etching) and/or anodization, in each previous study experimental parameters were completely different. In particular the material used for sandblasting, the nature of acids, the sequence of use, the difference in applied electrical potential, the treatment times are variable which have a significant effect on the final result [22,37]. The parameters used in the present study are unable to produce anatase or crystalline TiO_2_ on the surface of TA implants, but this finding does not conflict with the fact that other treatments have produced anatase or different surface morphologies.

For example, a recent study evaluated in vivo in the rabbit model Ti6Al4V implants treated by a thermal process to obtain a layer of anatase on the surface. The histological and histomorphometric results of that study showed a significant increase in bone–implant contact after treatment to obtain a surface layer of anatase [54].

In this study all surfaces subjected to sandblasting with Al_2_O_3_ had a surface morphology equivalent and similar to the analogous surfaces reported in the literature [22,37].

As previously observed [46], the results of this study confirm the presence of Al_2_O_3_ residues on the final surface after sandblasting with corundum. The experimental surface treatments used in this study, and in particular the acid etching treatment, are not able to completely decontaminate the implant surface. Other studies reported this event [12,42].

Furthermore, even the applied anodizing treatment did not change the surface morphology of the implant but only the crystallographic conformation of the titania.

Confirming previous studies, the anodizing treatment carried out on CP implants determined the growth of anatase. Unlike those previously published [40], the anodizing treatment used in this study did not result in a change in surface morphology, even at very high magnification (25 k×).

Furthermore, the results of our study indicate that anatase can also occur with less elaborate surface treatments than anodization, and at low temperatures.

The evolution of the surface is given by an increase in the thickness of oxide on CP and TA implants and by crystallization on CP implants.

In order to evaluate the possible formation of an ordered TiO_2_ surface layer during the manufacturing process, for each sample, the ratio O/Al has been calculated by using the atomic percentage values provided by EDS measurements.

The measured Oxygen percentage could have dual source. It could be due to (i) the formation of a Titanium oxide surface layer, as a product of the manufacturing process, or to (ii) the presence of residual corundum (Al_2_O_3_) micrograins as consequence of the sandblasting.

To better clarify this, the EDS analyzes have been carried out with 160× magnification in order to average as much as possible the presence of corundum micro-grains in the irradiated volume. Moreover, for each sample, the EDS measures have been focused on a single corundum grain, thanks to higher magnification, in order to evaluate the Oxygen percentage in the only corundum phase.

At this point it is important to underline that the Raman measurements evidenced a short-term stability of the anatase layer for CP3 and CP4 samples, as derived for repetition of the spectra in a few weeks. This stability accounts for mechanical manipulation and laser irradiation. Of course it will be highly important to evaluate long-term mechanical, chemical and phase stability in particular in view of the evaluation of the biological effects of the presence of crystalline TiO_2_ on the surface of dental implants. In fact, it is known that the presence of anatase and crystalline TiO_2_ can have biological effects, for example on osseointegration and antibacterial properties, but it is not known whether these effects, in turn, have a significant clinical effect. The results of this study show that it is possible to obtain a layer of anatase on implant surfaces without altering their surface morphology. Future studies should evaluate the biological significance of the presence of anatase by comparing morphologically identical surfaces differing only for the presence of anatase on the surface. This aspect will be the subject of future investigation.

## 5. Conclusions

In this work we, have studied by Raman microscopy the crystalline ordering of implant surfaces depending on starting material and surface machining and treatments commonly used for dental implants. Regardless of starting material, titanium-alloy or commercially pure titanium, the increase in the complexity of the surface treatments determines an increase in the thickness of titania on the surface of the implants. However, only for CP implant did the treatment by Al_2_O_3_-blasting and HNO_3_/HF etching produce a well ordered anatase layer, further increased by the anodization treatment. On the contrary a disordered oxide layer was obtained from implants made of titanium alloy, even for anodized samples. The amount of oxide has been also corroborated and confirmed by the SEM-EDS analyses. It is important to note that the treatments used in this study allowed the formation of anatase also in the absence of high temperatures and without anodization. The observed oxide layer formation highlighted by Raman and EDS spectra is accompanied by a substantial invariance of the surface morphology of the implants.

## Figures and Tables

**Table 1:** Composition of cp grade IV Titanium and Titanium alloy. Weight percent.

Bulk Material	N	C	H	Fe	O	Al	V	Ti
Cp grade IV	0.03	0.10	0.015	0.05	0.40	_	_	balance
Ti-6Al-4V alloy	0.05	0.08	0.015	0.30	0.20	5.50–6.75	3.50–4.50	balance

**Table 2:** List of analyzed samples and respective identification codes.

Code	Bulk Material	Surface Treatment
TA1	Ti-6Al-4V alloy	Machining
CP1	CP Titanium	Machining
TA2	Ti-6Al-4V alloy	Al_2_O_3_-blasting
TA3	Ti-6Al-4V alloy	Al_2_O_3_-blasting and HNO_3_/HF etching
CP3	CP Titanium	Al_2_O_3_-blasting and HNO_3_/HF etching
TA4	Ti-6Al-4V alloy	Al_2_O_3_-blasting, HNO_3_/HF etching and anodization
TA4p	Ti-6Al-4V alloy	Al_2_O_3_-blasting, HNO_3_/HF etching, anodization and cold plasma
CP4	CP Titanium	Al_2_O_3_-blasting, HNO_3_/HF etching and anodization
